# A detection and configuration method for welding completeness in the automotive body-in-white panel based on digital twin

**DOI:** 10.1038/s41598-022-11440-0

**Published:** 2022-05-13

**Authors:** Hao Li, Bing Li, Gen Liu, Xiaoyu Wen, Haoqi Wang, Xiaocong Wang, Shuai Zhang, Zhongshang Zhai, Wenchao Yang

**Affiliations:** 1 Key Laboratory of Intelligent Manufacturing of Mechanical Equipment, Zhengzhou University of Light Industry, Zhengzhou, 450002 China; 2 University of Greenwich, Park Row, London, SE10 9LS UK; 3 Tianjin Miracle Intelligent Equipment Co. LTD, Tian Jin, 300131 China

**Keywords:** Mechanical engineering, Electrical and electronic engineering

## Abstract

To address the problems of poor welding completeness and inefficient configuration for defective automotive body-in-white panels, we propose a method for detecting and configuring the welding completeness of automotive body-in-white panels based on digital twin (DT) and mixed reality (MR). The method uses DT to build an MR-oriented DT framework for the detections and configuration of body-in-white panel welding completeness. We propose a method to build a DT knowledge base for panels, a Yolov4-based welding completeness detection method, and a MR-based configuration method for the welding completeness in panels. Our team develop a panel welding completeness detection and configuration system to fully validate the effectiveness of the method.

## Introduction

With the rapid development and application of a new generation of information technology, the global manufacturing industry is accelerating toward digitalization, networking, and intelligence. Intelligent manufacturing is receiving more and more attention from various countries^1^. The welding completeness of the body-in-white panels, as the main component of the vehicle, is the key to the efficiency of the body-in-white production. In order to meet the process requirements, bolts, nuts, locating pins, and other types of parts need to be welded onto the specific hole positions of some panels before they are fused into body-in-white parts. However, due to the large variety of panels and small volume of welded parts, it is easy to omit welded parts only by workers' judgment and manual welding, resulting in the difficulty of ensuring the welding integrity of panels. At present, there are limitations in the means of body-in-white panel welding completeness detection and configuration, mainly in two areas:

1. There is a lack of an efficient method for detecting the completeness of panel welding. First, manual detection is still widely-used, but there is a great deal of uncertainty and requires much labor input. In addition, when using the traditional proximity sensor for detection, the sensor is easily damaged, resulting in low detection efficiency and low accuracy.
2. The configuration method of panel welding completeness needs to be improved. At present, the guidance process mainly relies on two-dimensional drawings, while results in 2D graphics are not intuitive.

As a new method of intelligent manufacturing application, Digital Twin(DT) is to create the virtual model of a physical entity digitally, through two-way mapping and real-time interaction between physical entities and virtual models, and to achieve optimal design of industrial products, production line planning simulation, manufacturing process optimization, and service operation control^2^. The concept of DT was first proposed by Professor Grieves at the University of Michigan in 2003 in a PLM (Product Lifecycle Management) class^3^. DT has been used in more than 40 aerospace, medical, smart city, smart building, and automotive manufacturing^4–7^. Mixed Reality (MR) can achieve virtual-real integration data interaction and assist in decision optimization through real-time data collection, scene construction, and virtual-real registration^8^. Fusion detection methods based on DT and MR can provide digital and intelligent support for body-in-white panel welding completeness detection and configuration, effectively improving the welding efficiency of body-in-white panels.

This paper proposes a DT-based method for detecting and configuring welding completeness of body-in-white panels. Based on the way, our team develops a system that achieves physical-information fusion and visual interaction in panel welding completeness detection and configuration, which improves the efficiency of body-in-white welding. The paper’s overall structure is as follows: Sect. 1 summarizes the research by scholars on digital twin shop intelligent production lines and MR-based workpiece quality detection. Section 2 gives a framework for detecting and configuring the welding completeness of body-in-white panels based on DT. Section 3, a knowledge base construction method for the DT of body-in-white panels, and a panel welding completeness detection method are proposed. Section 4 validates the proposed approach through typical cases and provides a comparative analysis. Section 5 summarizes the study and provides directions for future research.

## Related work

### Review of intelligent production line research in digital twin shops

The digital twin for intelligent production line on the shop floor is one of the current research hotspots of digital twin technology. Tao et al.^9^ proposed the concept of a digital twin workshop in 2017. Based on this, Leng et al.^10^ discussed the basic theory and key technology of workshop information physics systems driven by the digital twin. Many scholars then began to explore digital twin applications on the shop floor, mainly focusing on modeling and simulation of shop floor production lines^11,12^, scheduling optimization^13,14^, real-time visual monitoring^15,16^ and production quality control^17,18^. Theoretical development has driven the rapid application of digital twin technology in the intelligent production line of the workshop. In the automotive manufacturing workshop, Liu et al.^19^ applied digital twin technology in the automotive remanufacturing workshop to achieve resource recycling. Leng et al.^20^ verified the remote near-physical debugging method based on digital twin through the smartphone assembly line to solve the compatibility problem of the production line equipment. Li et al.^21^ proposed a digital twin modeling method for body-in-white welding lines to shorten production line setup and commissioning time. Dou et al.^22^ used digital twin technology in a panel furniture production line personalized custom design to shorten the design cycle. Son et al.^23^ designed a digital twin-based CPS system to predict the body line's capacity and determine the scheduling plan's feasibility. To sum up, digital twin technology has been applied in the design or operation of workshops and production lines, which significantly affects production efficiency and the intellectual level of workshops or production lines.

### Review of MR-based workpiece quality detection research

The technology-based DT and MR have progressed in auxiliary maintenance, education and safety training, human–computer interaction and cooperation, and intelligent building. However, most relevant research remains theoretical, and the correct implementation still faces some challenges^24^. For example, Zhang et al.^25^ constructed the auxiliary maintenance guidance system of DT mining machinery equipment based on MR equipment. Still, the poor accuracy of virtual and realistic registration led to low maintenance efficiency. There are few kinds of literature on workpiece quality inspection based on DT. Traditional manual detection means are not sufficient to meet the requirements of efficient and accurate quality detection of workpieces. At the same time, MR-based workpiece detection methods have the characteristics of solid information visualization and high detection freedom to meet the demand for real-time workpiece detection. With this in mind, MD Mura et al.^26^ developed a system for MR detection of automotive body panels, which can efficiently assist workers in detecting panel gaps and reducing flushness errors. Liu et al.^27^ combined a DT machining system with MR, which can detect the quality of machined parts on machine tools in real-time. The accuracy of workpiece quality detection mainly depends on the detection precision of vision algorithms. In recent years, vision-based defect detection algorithms have developed rapidly in automotive workpiece detection. For example, Wang et al.^28^ applied deep learning algorithms in MR systems for quality detection of automotive assemblies. Zhou et al.^29^ developed an automatic vision detection system for detecting automotive door surface defects. Currently, related research has been extended to safety belt quality, body coating quality, and body assembly quality detection^30,31^. Overall, MR-based workpiece quality detection techniques were initially applied to automotive parts detection, but there is still a lack of efficient methods to ensure the completeness of panel welding.

## System framework

**Figure 1:**
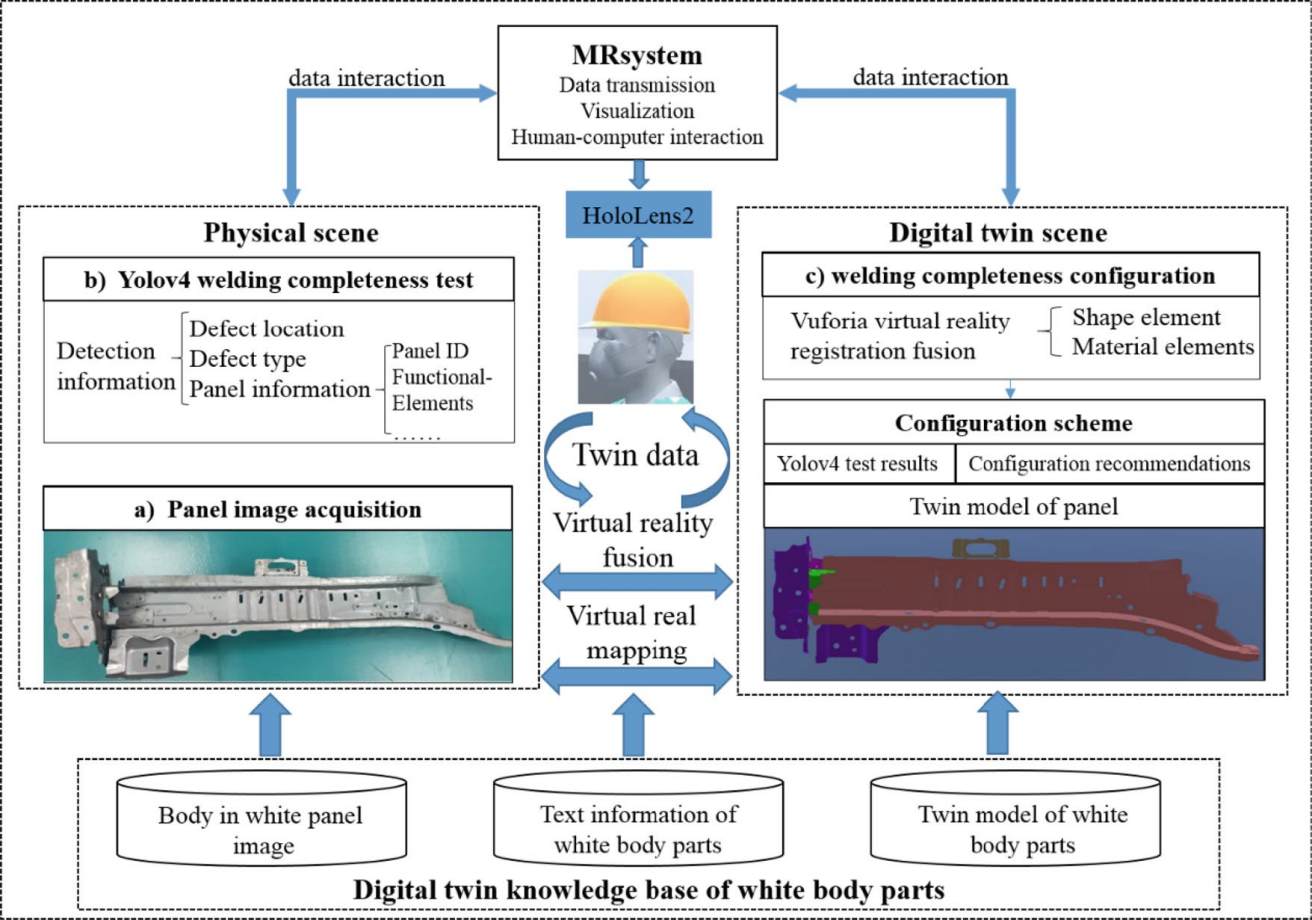
Detection and configuration framework for welding completeness of body in white panels based on digital twin.

A framework for a digital twin-based body-in-white panel weld integrity detection and configuration system is proposed for the weld integrity detection and configuration problem. As shown in Fig. 1, the framework consists of a physical scene, a digital twin scene, and an MR system. First, using digital twin technology, the MR system obtains the panel welding completeness information from the physical scene and passes the panel twin data into the digital twin scene. Second, the corresponding panel welding completeness configuration scheme is matched from the body-in-white panel digital twin knowledge base. Lastly, the mapping and data interaction between the physical and twin scenes are realized.

The DT-based body-in-white panel welding completeness detection and configuration flow are shown below.

1. Panel image acquisition. The HoloLens2 device is used to capture the body-in-white panel image or video data in the physical scene and transfer the data to the server via HTTP protocol.
2. Yolov4 welding completeness detection. First, the trained Yolov4 detection algorithm is deployed to the server. Secondly, welding completeness detection is performed on the collected real-time information of the panel. The detection information includes defect type, location, and corresponding reliability. Finally, the detection results are converted into image and text information and transmitted to the configuration plan in the digital twin scene, guiding the panel configuration repair.
3. Welding completeness configuration. First, in the twin scene, the shape and material elements of the physical panel in the MR field of view are analyzed. Secondly, the Vuforia-based virtual and real registration fusion methods are used to match the welding completeness configuration scheme from the body-in-white panel digital twin knowledge base. The configuration scheme includes the panel twin model, the detection result of Yolov4, and the corresponding repair suggestion information. Finally, with the help of the MR system, the configuration scheme is displayed on the physical objects of the connected body-in-white panels to guide the operator to perform quick configuration tasks.

This paper adopts the yolov4 target detection algorithm to realize the automatic detection of panel welding completeness, which speeds up the detection speed of workers. At the same time, this paper uses the virtual and real registration method to match the configuration plan from the DT knowledge base, strengthening the digital and intelligent guidance capabilities of the method and facilitating the operator to repair the defective panel quickly.

## Method implementation

### DT knowledge base construction technology for body-in-white panels

**Figure 2:**
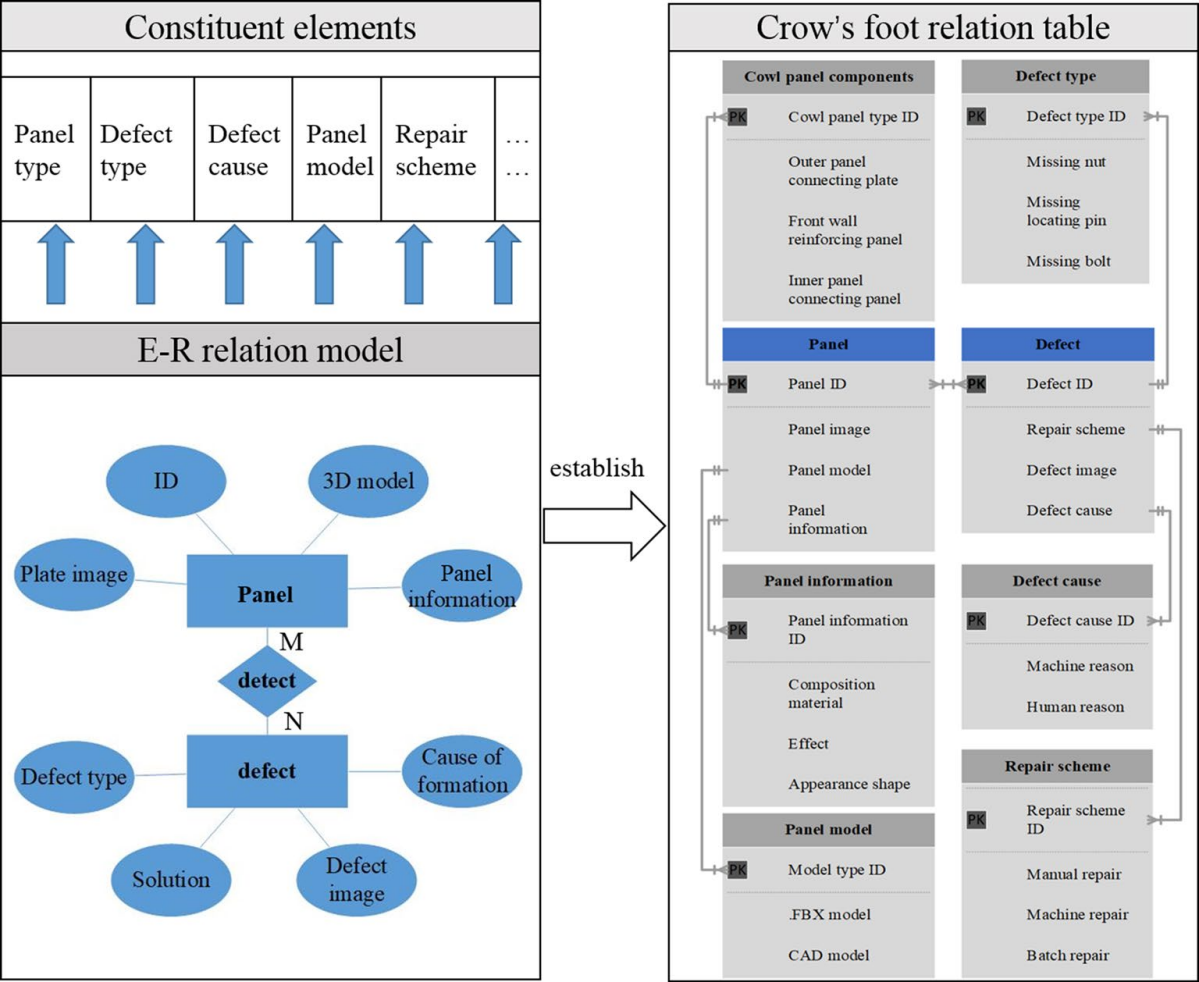
Construction of body in the white digital twin knowledge base.

Constructing a DT knowledge base for body-in-white panels facilitates the detection system to analyze panel welding completeness issues from a more professional perspective. Moreover, it can provide operators with professional configuration solutions, which can be used to complete the configuration of panels and parts efficiently. Compared with textual knowledge bases composed of textual semantic data, graphical knowledge bases are more advantageous in terms of knowledge structure expression and logical reasonin^32^. Based on this, this paper adopts the form of a graphic knowledge base to construct a knowledge base for body-in-white panel welding completeness detection and configuration problems. As shown in Fig. 2, the visual knowledge base mainly comprises entities, entity relationships, and attributes. The E-R knowledge base relationship model is established based on attribute components such as panel type, defect type, and cause. Specifically, the panel and defect are regarded as the entity, detection as the entity-relationship, and the connection of two entities, as many-to-many relationship M: N. Among them, the panel entity contains the panel ID, virtual model, panel image and panel information, etc. The defect entity has the defect type, formation cause, defect image, solution, etc. In addition, Crow's foot data relationship table is established based on the E-R relationship model. In this paper, we take the example of a body-in-white dash panel component and analyze what is contained under the attributes of the panel and the defective entity. First, a relational database is established based on the Crow's foot data relationship table to form a digital twin knowledge base for body-in-white panel parts. Then, the MRTK resource package was imported into Unity 3d software, and scripts were written to call the knowledge base's API. Lastly, the app is deployed to HoloLens2, using this knowledge base to guide the operator for configuration and repair.

The knowledge base guidance program can be summarized: First, the two entities, panel and defect, provide information in pictures, models, scene files, etc. The combination of information is formulated based on the E-R relationship model and presented on the HoloLens2 MR device. Then, the operator can get the ID number of the panel, the virtual model, the standard image of the panel, the type of defect, the cause, and the solution related to the defect entity of the panel in the system. Finally, the operator can obtain standardized and intelligent service from the system, which improves the efficiency of configuring panel welding completeness.

### Yolov4-based welding completeness detection method for body-in-white panels

#### Yolov4 algorithm

**Figure 3:**
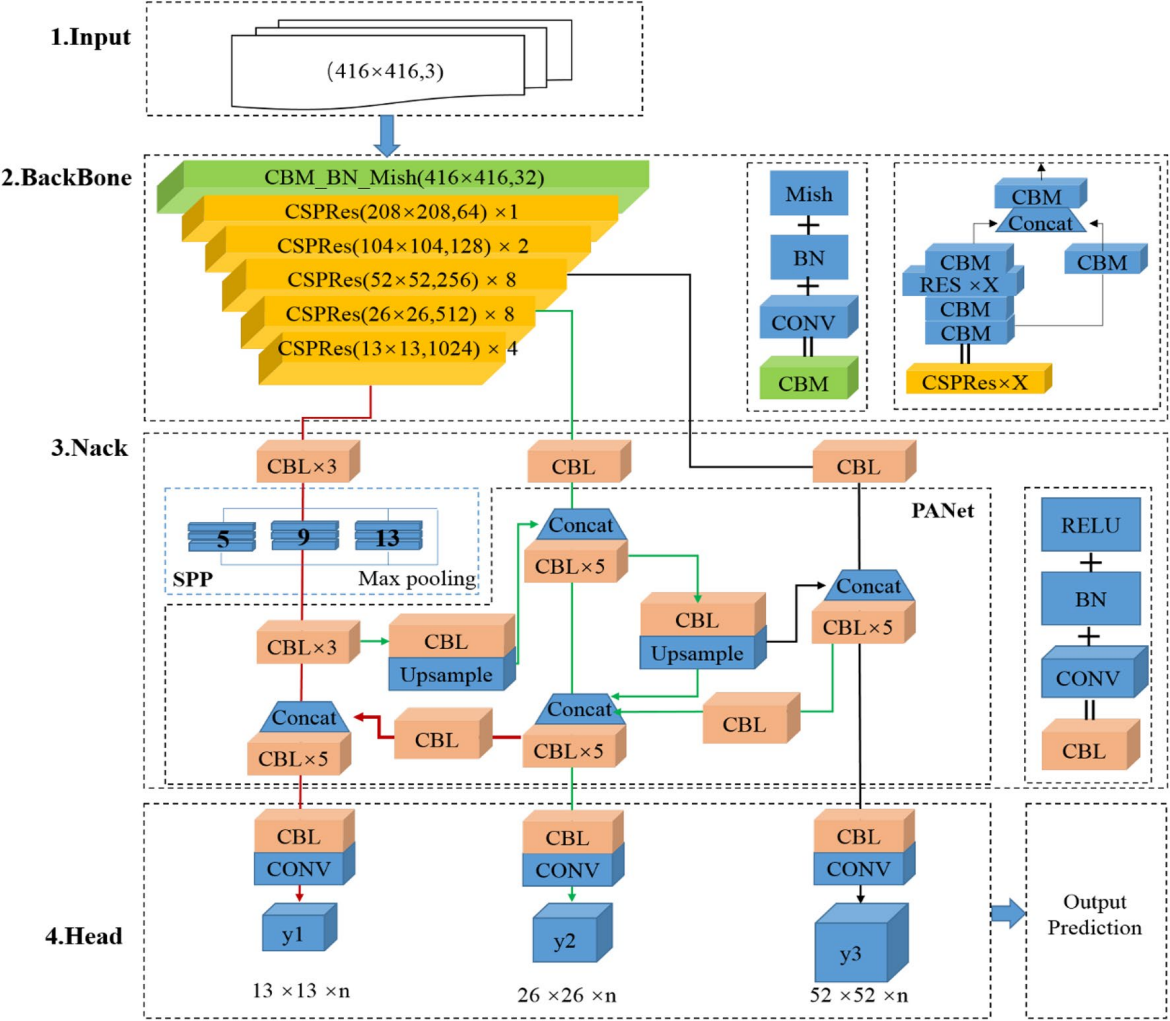
Yolov4 network structure.

This paper uses the Yolov4 target detection algorithm to detect the welding completeness of body-in-white panels. Compared with the traditional impanel matching algorithm, the former has stronger feature extraction and learning ability and more incredible detection speed and accuracy advantages.Yolov4 is a target detection algorithm proposed by Alexey Bochkovskiy et al.^33^, and compared to YOLOv3, the former makes optimizations in terms of the main feature extraction network, feature pyramids, and training tips^34^. The flow of the algorithm is shown in Fig. 3.

The algorithm sets the size of the image at the input. Since the down-sampling parameter is 32, the size of the input image is a multiple of 32.

The backbone network uses the CSPDarknet53 method. The method uses the Mish activation function to aggregate image features. In addition, the method optimizes the gradient information to reduce the number of model parameters and improve the model inference speed. Meanwhile, the Drop-Block regularization method extracts image features to improve the model generalization ability and prevent overfitting. Moreover, the Mish activation function is smooth. It has no positive boundary, passing the image information into the network more entirely and improving the model accuracy and generalization ability. The formula of Mish activation function is as follows:

1 $$y\_mish = x \times \tanh \left( {\ln \left( {1 + e^{x} } \right)} \right)$$

The neck feature pyramid is used to connect the backbone network, which uses the SPP module, and the FPN + PAN method to extract and fuse the image features and pass them to the Head prediction layer. In particular, the SPP module uses four pooling sizes of 1 × 1, 5 × 5, 9 × 9, and 13 × 13. The pooling operation increases the perceptual field and separates the most significant contextual features. The FPN method iteratively extracts the semantic and path features from the image in a top-down and bottom-up manner, respectively. The PAN instance segmentation algorithm adds bottom-up paths, fusing feature information of different sizes and output feature maps of three scales.

The head is predicted for the image features of the neck. First, a K-means clustering algorithm is used to obtain the test frame and assign it to the feature map. Then, the test frames, confidence levels, and categories are decoded in turn. Finally, the DIOU_NMS (non-maximal value suppression) algorithm filters the prediction frames that satisfy the confidence threshold and output the prediction results. Where, the prediction result = detection frame position + detection confidence + label category. the DIOU_NMS method considers both the IoU (intersection and merging ratio of detection frames to real frames) and the center distance of two detection frames to accelerate the loss function convergence speed^35^. The DIOU_NMS formula r is as follows.

2 $$S_{i} { = }\left\{ {\begin{array}{*{20}c} {S_{i} ,} & {IoU - R_{DIoU} (\mu ,B_{i} ) < \varepsilon } \\ {0,} & {IoU - R_{DIoU} (\mu ,B_{i} ) \ge \varepsilon } \\ \end{array} } \right.$$

where $S_{i}$ denotes the confidence value of the category, $B_{i}$ is the set of all prediction boxes in the class, $\mu$ is the one with the highest confidence and $B_{i}$ is the largest confidence level, and $\mu$ is the screening threshold (artificially set)

3 $$R_{IoU} = \frac{{\rho^{2} (b,b^{gt} )}}{{c^{2} }}$$

where $R_{{I{\text{o}}U}}$ denotes the canonical term of the *DIoU* loss function. $\rho$ is the Euclidean distance, $b$ and $b^{gt}$ are the center coordinates of the two predictor frames. $c$ refers to the diagonal length of the minimum external matrix of the two predictor frames.

#### Dataset generation

1. Image acquisition. The Yolov4 algorithm in this paper detects panel scenes acquired while the operator is wearing HoloLens2 on his head. The quality elements of the image or video, such as sharpness, focus, and noise, depending on the HoloLens2 device. The image or video sample captured by HoloLens 2 shall reflect important feature information, such as panel shape, surface hole location, nuts, bolts, locating pins, etc. Among them, the single-shot pixel of the HoloLens2 device is 8 million, and the video quality is 1080p 30fps.
2. Data pre-processing. First, the video samples are collected, and the frames are segmented into a single image at 5 fps intervals. Secondly, the blurred images and panel shape mutilated images are screened and eliminated. Lastly, the welding completeness of each panel is classified again. According to the size and shape of different panel holes, the samples are classified into 6 cases of nuts, bolts, locating pins with missing nuts, missing bolts, and missing locating pins. The training model adopts the data set format of VOC, including original images and label files. The number of original pictures is 1100. The data set is made and divided into the training set and test set in the proportion of 9:1. Therefore, 990 training set images and 110 test set images are finally obtained.
3. Data labeling. The labeling software was used to manually label the images in the training set data. The nuts, bolts, locating pins, and corresponding missing cases were marked to obtain the label file. The designed label categories are shown in Table 1.

**Table 1:** Label categories.

Serial number	Label category
1	Nut
2	Bolt
3	Locating pin
4	Missing nut
5	Missing bolt
6	Missing locating pin

#### Model training

1. Model training. Here is the information on experimental environment: Window10, Intel(R) Core(TM) i7-8700 CPU @3.20GHZ processor, RAM 16G, graphics card NVIDIA GTX1060, Python3.7. Model parameters are set as follows. Input image size is 416 × 416. The batch is set to 4, and the label smoothing is set to 0.05. The breakpoint continuation training method is adopted. One breakpoint is set every 350 times, four breakpoints are set, 350 weight files are generated after 350 times of training, and the best weight file is manually selected as the initial weight of the next breakpoint. The total number of training times is 1400 times. During the test, the confidence is set to 0.4, and IOU is set to 0.4.
2. Evaluation metrics. In this paper, we mainly evaluate the effectiveness of model training in terms of detection accuracy and efficiency. The evaluation metric used is the mean Average Precision (mAP), the average detection accuracy AP of all categories, and the number of image frames per second FPS detected by the algorithm.

### MR-based method for body-in-white panel welding completeness configuration

The operator is immersed in the MR environment with a HoloLens2 device on his head. The system in this paper uses a Vuforia-based virtual-real fusion approach, matched with a corresponding digital twin guidance solution, combined with human–machine interaction to achieve efficient guidance for the operator.

#### Virtual real registration fusion based on Vuforia

Virtual-real registration fusion technology is the key to achieving virtual and physical fusion interaction. It is mainly divided into virtual reality registration fusion based on machine vision, sensor, and hybrid virtual reality registration fusion^36^. In this paper, the scene is obtained by calling the hololens2 camera. Therefore, the virtual reality registration fusion technology based on machine vision is adopted. The machine vision-based virtual-real registration fusion technique is divided into two types with and without artificial markers. The anchoring methods and characteristics are shown in Table 2. The artificial marker-based virtual-real registration fusion technique requires a physical marker or a virtual marker attached to the detection target for anchoring the virtual model. The virtual model can be anchored by scanning QR codes, circular codes, or holograms. This method can achieve high registration accuracy and stability. Since the object of this paper is applied in a multi-species and multi-batch detection scenario, this method of attaching a logo code to each panel is less efficient and needs to consider situations such as easy destruction of the logo. The hologram anchoring method is a virtual-real registration fusion by matching a virtual identifier with a physical object. The method is essentially the same as the manual marker method, except that a virtual marker replaces the physical marker. In addition, the method can be adapted to mobile scenarios and small part registration scenarios. In comparison, the feature matching method with no logo has stronger robustness. However, there are also problems such as easy loss of registration targets and errors.

**Table 2:** Virtual reality registration method based on machine vision.

Type	Anchoring mode	Characteristic
Manual identification method	QR code	The registration accuracy and stability are high, and the entity identification needs to be attached, suitable for fixed scenes
	Circular code	
	Hologram	The registration accuracy and stability are high, and the virtual logo needs to be attached, which is suitable for mobile and small target registration scenarios
Unmarked method	Feature matching	Registration is flexible and convenient, without identification, and is suitable for a wide range of scenarios

**Figure 4:**
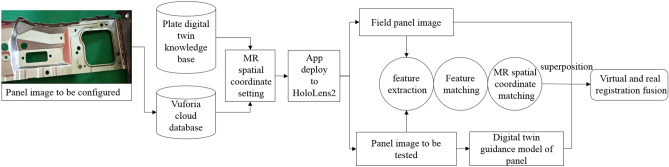
Virtual real registration fusion process based on Vuforia.

Vuforia-based virtual and real registration fusion method is adopted in this paper, considering the complex and changeable detection environment and the variety of detection panels.The method is a feature matching anchoring method without a marking method. Vuforia SDK is an AR toolkit developed by Qualcomm, and the core of its algorithm is to match and track the target by a feature point matching algorithm. The flow of the Vuforia-based virtual-real registration fusion method is shown in Fig. 4. Firstly, the standard panel image to be detected is uploaded to the Vuforia cloud database, and the data file is exported to Unity3d software. Secondly, the standard panel is set with the corresponding guide model in the digital twin knowledge base for MR spatial coordinates to determine the display position. Thirdly, the project app is deployed to HoloLens2, and the live panel images are acquired by calling the HoloLens2 camera. Finally, the image is matched with the panel inspected for feature point extraction, feature matching, and MR coordinate matching. The virtual panel digital twin knowledge base scene is overlaid with the live image to combine virtual and real registration and guide the operator in the configuration.

#### Human–computer interaction function construction

The good human–computer interface and function settings can assist the operator in configuring the repair of defects in body-in-white welded panels. When the operator wears a HoloLens2 device on his head, he views the panel for key information through gestures, voice control, and other interactive operations. The main functions are as follows.

1. Basic interaction functions. By importing the Mixed Reality Toolkit (MRTK) resource package in unity3d, we designed the GUI operation panel with basic functions such as gesture operation, line of sight tracking, and voice control to meet the basic interaction operation needs of the operators.
2. Scene real-time acquisition. We designed the UI for taking photos, which allows the operator to take an image or video of a detected live scene by invoking the HoloLens2 camera.
3. Abnormal information is reported. When novice operators wear HoloLens2 equipment to test the completeness of plate parts, the batch plate parts have some abnormal conditions such as more defects and are difficult to repair, and can collect real-time scene video with the help of the camera on Hololens2. The video or picture data will be transmitted to the workshop quality control server-side. The computer will report the abnormal situation. In this way, the quality control personnel will get the batch abnormalities using voice video conversation on PC or cell phone mobile terminal, and then call the relevant personnel to dispose of the batch abnormal panels online. It should be noted that the human–computer interaction interface for abnormal information reporting includes modules such as HTTP communication connection, voice call, and video connection.

## System development and validation results

This paper developed a panel welding completeness detection and configuration system for body-in-white dash panel connection components. The technology is verified in 3 aspects: detection environment sensing and acquisition, Yolov4 welding completeness detection, and welding completeness configuration.

**Figure 5:**
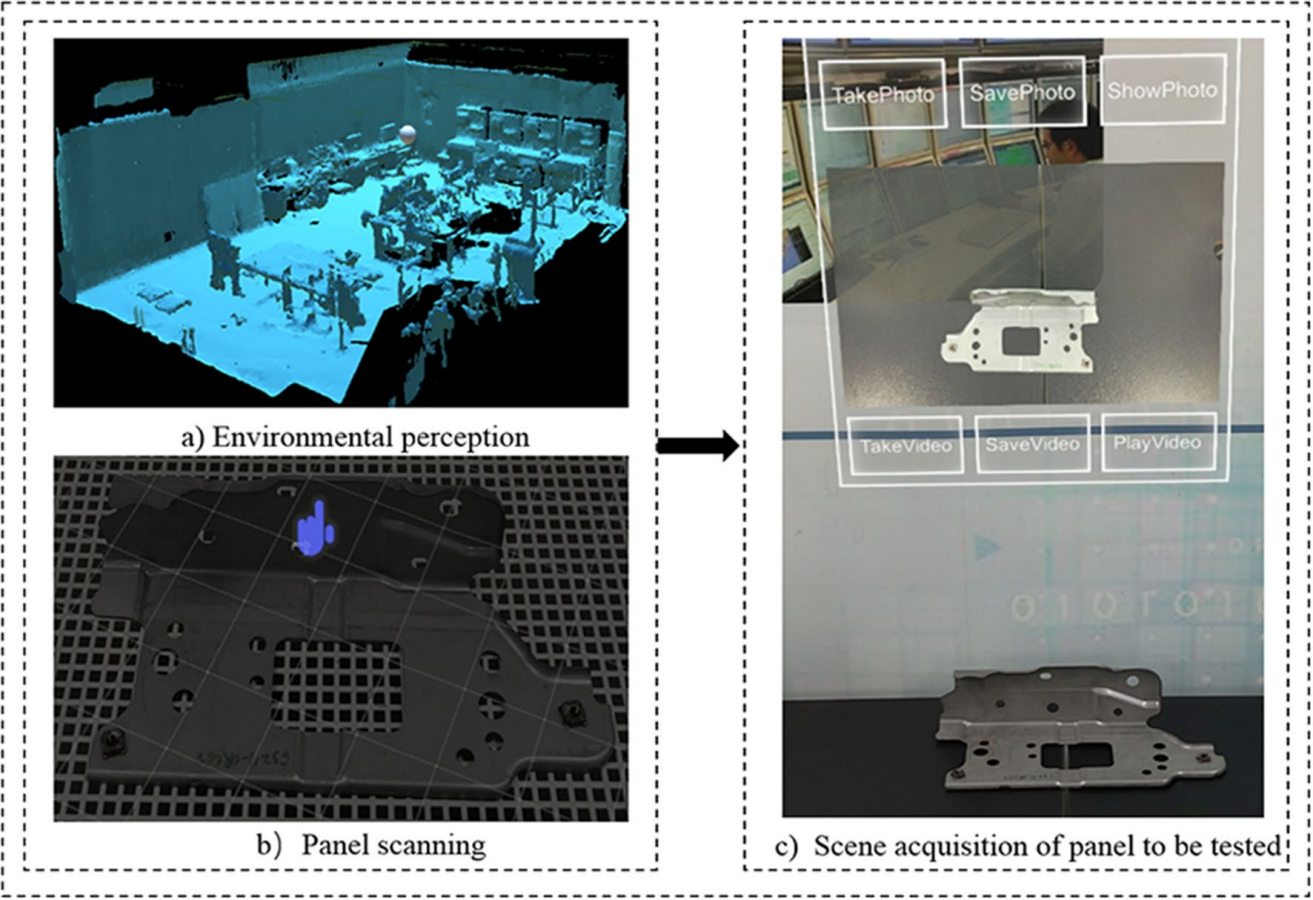
Environment perception and acquisition.

**Figure 6:**
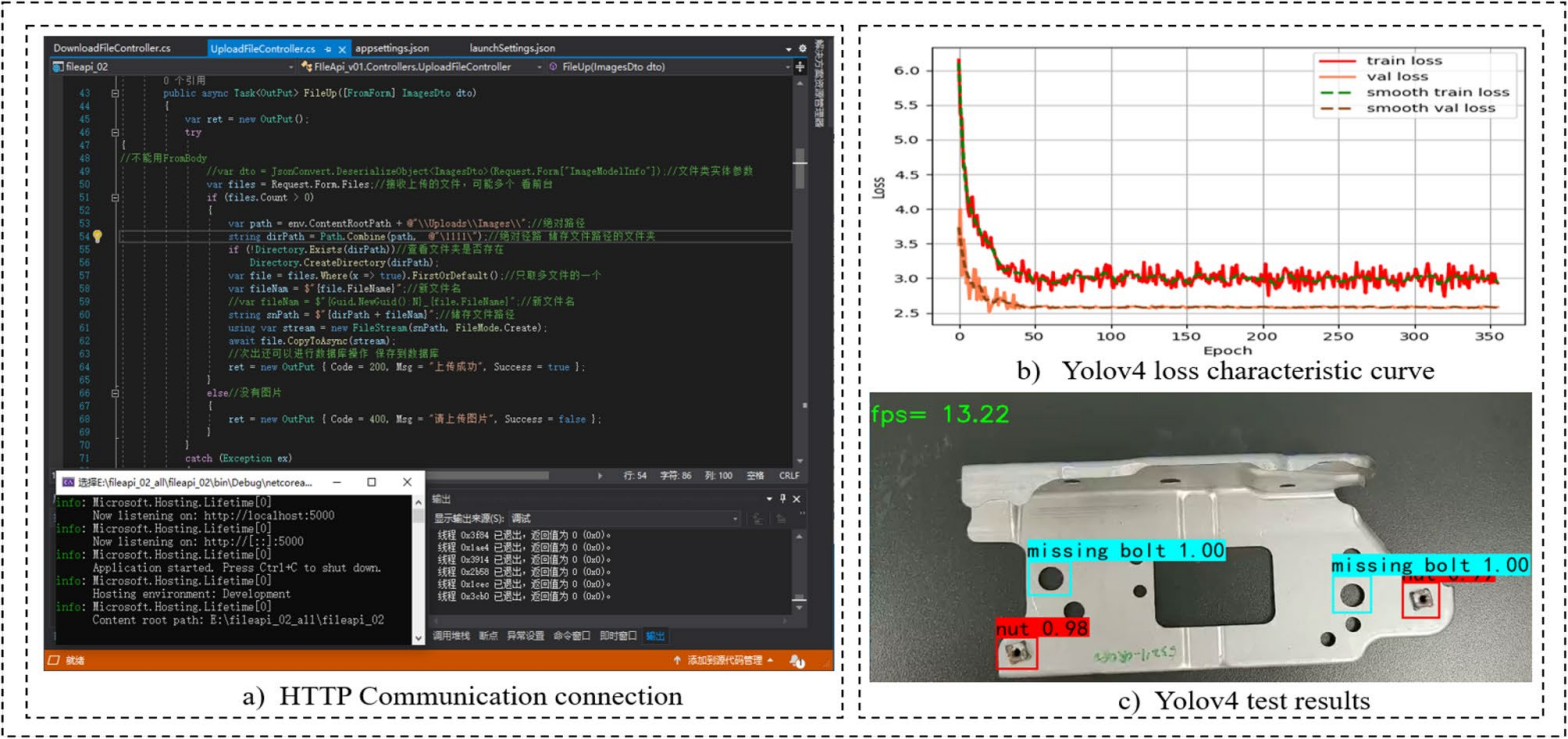
Welding completeness detection based on yolov4.

**Figure 7:**
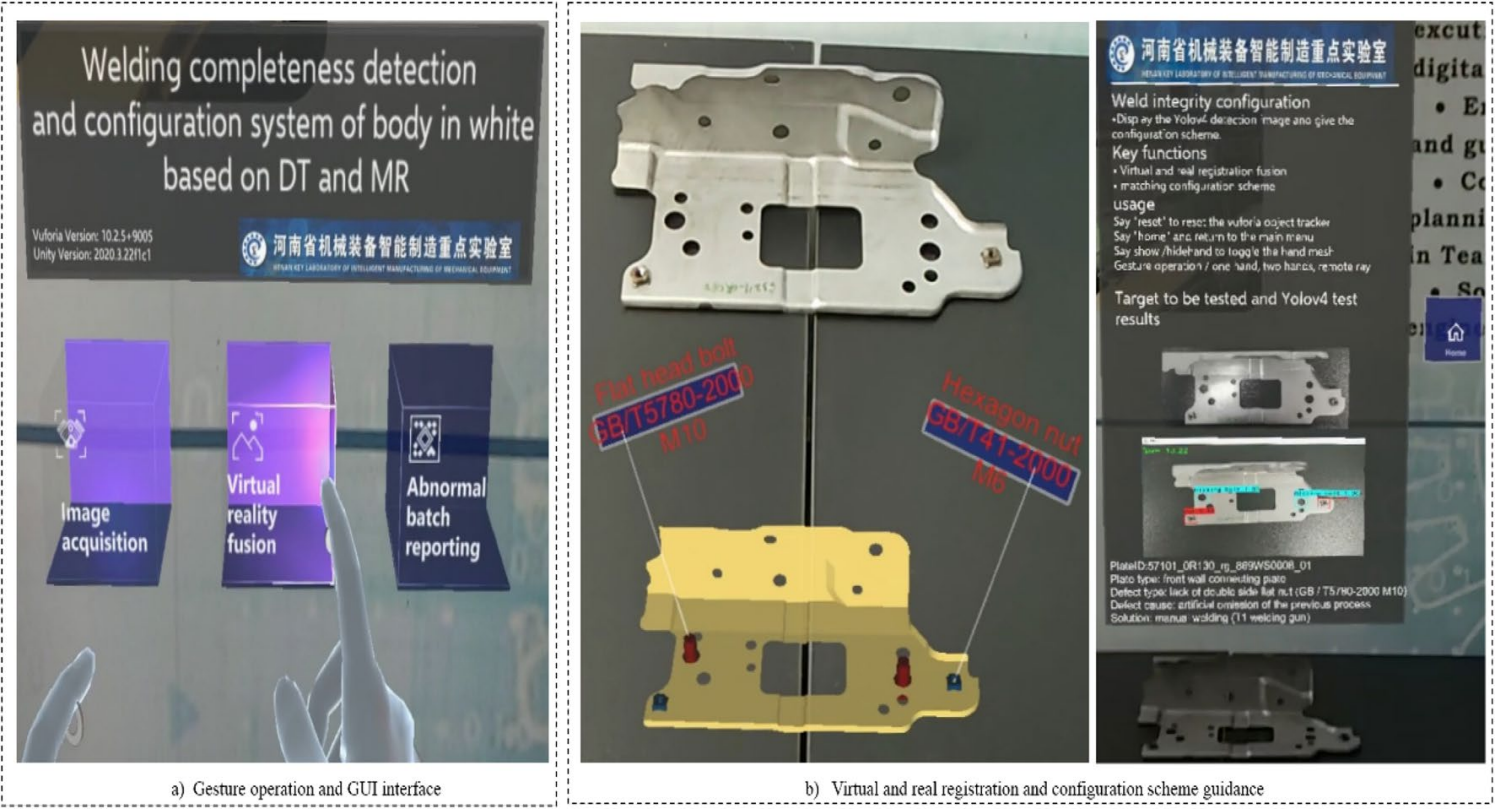
Panel welding completeness configuration.

1. Environment perception and image acquisition. As shown in Fig. 5. First, the operator wears HoloLens2 glasses and enters the system APP. Then, the system senses the detection environment by initializing the scanned inspection scene and creating a 3D model. The panels to be detected are scanned in fine detail. Finally, the operator clicks on the image acquisition UI to capture images of the panel scene in real-time.
2. Panel welding completeness detection. Firstly, the image and video data captured by the HoloLens2 device is transmitted to the PC server in fast real-time using the HTTP wireless communication protocol. Then, the Yolov4 image processing module starts the detection and returns the detection results to the device to achieve fast detection. As shown in Fig. 6, serial number (b) is the loss feature curve during the training of the Yolov4 model. It is worth mentioning that the model converges completely when the number of iterations is set to 1400, and the val_loss value is stable at about 2.5, indicating that the model fits well. Finally, the images and videos from the test set are selected to test the model. The model training detection accuracy mAP reached 90.1%, FPS value can go 13.22 frames / S. The training model has good detection accuracy and high detection efficiency. Moreover, it can complete the detection task of panel welding completeness. Serial number (c) is the detection result of the front surround panel component connection panel. It shows that two nuts are intact and two bolts are missing in the four holes, and the system operation detection frame rate in HoloLens2 is 30 frames / s.
3. Panel welding completeness configuration. When configuring the defective panel (as shown in Fig. 7), the operator faces the system GUI operation interface, enters the corresponding module through gestures, gaze tracking, and voice, and triggers the corresponding function button to realize essential human–computer interaction operation. When encountering difficult problems to configure and handle, the abnormal situation can be reported or applied for expert assistance for online guidance. The HoloLens2 device scans the panel , matches the model in the digital twin knowledge base and displays the virtual model at the preset MR spatial coordinates to achieve virtual-real registration fusion. A quick virtual-real comparison is made to clarify the missing parts according to the color markings of key parts on the virtual model. At the same time, the virtual model can be scaled and rotated accordingly to enhance the understanding of the panel structure and improve configuration efficiency.
4. Comparative experiment. Table 3 compares the traditional manual detection and configuration method and the method in this paper. Compared with the conventional method, this method can realize MR visualization, analyze the causes of panel defects, and recommend relevant configuration schemes.

To evaluate the adaptability of this method in practical application, five kinds of defective panels are selected for welding integrity detection and configuration experiment, in which the personnel participating in the experiment are unaware of the panel defects. The experimental conditions are divided into experienced, inexperienced, and worker + this system. The workers complete the panel detection and repair tasks under the guidance of the technical instructions. In this paper, the system operates according to the guidance of MR equipment and counts the average time required and the average number of errors.

It is hereby declared. This experiment was approved by the Key Laboratory of Intelligent Manufacturing of Machinery and Equipment in Henan Province. The experimental programs were carried out in accordance with the guidelines and regulations of the Key Laboratory of Intelligent Manufacturing of Machinery and Equipment in Henan Province. All participants of the experiment are aware of, and agree to participate in, this experiment. The experimental results are shown in Table 4. It can be seen that the detection and configuration system based on digital twin and MR can realize automatic detection and auxiliary visual configuration in the vision of workers and improve the detection efficiency and accuracy of inexperienced workers. However, in the scenario described in this experiment, whether experienced workers adopt this system because they are thoroughly familiar with the detection task of each panel. It is not significant to improve the detection efficiency and even interfere with the tasks of skilled workers. However, the development method in this paper still has advantages in improving the accuracy of configuration. The experiment also shows that the practical application effect of MR equipment is related to the complexity of the panels to be tested and the proficiency of workers, especially for the workers with insufficient on-site operation experience; its automatic detection and visual auxiliary effect are obvious.

**Table 3:** Comparison of main indexes between traditional manual method and this method.

Content	Artificial method	Paper method
Technical requirements for completeness of panel welding	√	√
The physical state of the panel	√	√
MR visualization	×	√
Panel defect analysis	×	√
Panel configuration suggestions	×	√
Exception reporting and remote expert assistance	×	√

**Table 4:** Performance test of manual method and this method.

Experience group	Detection and configuration average time/s	Detection or omission average/time
Experienced workers	145	0.4
Worker + text system	172.5	0.2
Inexperienced worker	230	0.8

## Conclusion

The welding completeness of the body-in-white panel is one of the critical factors affecting the welding efficiency of the panel. However, there is little research in this field in the past literature. In practical application, it only relies on manual detection and configuration of the welding completeness of the panel. The detection accuracy and configuration efficiency are low. It is often reworked due to the omission of nuts, bolts, and other components, resulting in losses. This paper presents a digital twin-based method for detecting and configuring welding completeness of automotive body-in-white panels to solve this problem. Based on this method, a corresponding system has been developed. Through validation, we have shown that the system effectively detects the welding completeness of the dash panel connections and guides the operator in repairing defective panels. In addition, comparative experiments have shown that this system effectively improves the efficiency and accuracy of detection by inexperienced workers. In summary, this paper presents a deep fusion of the physical inspection space of welded body-in-white panels with twin models and a bidirectional mapping of inspection data to configuration information. This method can be a positive reference for applying digital twin technology in the MR field.

Panel welding completeness detection and configuration is a long-time repetitive work in the workshop site. The intervention of MR equipment will improve the accuracy of workers' detection and configuration. At present, this method still has shortcomings. For example, using HTTP protocol may lead to slow transmission of video data stream and jamming. In the next step, the communication technology between HoloLens2 and PC will be studied to improve the fluency of system application.
